# Aripiprazole in the real-world treatment for irritability associated with autism spectrum disorder in children and adolescents in Japan: 52-week post-marketing surveillance

**DOI:** 10.1186/s12888-021-03201-6

**Published:** 2021-04-22

**Authors:** Yuna Sugimoto, Kayo Yamamura, Tomoyo Takayama, Yasuhiko Fukuta, Kazuo Aoki, Katsunaka Mikami, Akemi Tomoda

**Affiliations:** 1 Pharmacovigilance Department, Otsuka Pharmaceutical Co., Ltd., Tokyo, Japan; 2 Medical Affairs Department, Otsuka Pharmaceutical Co., Ltd., Tokyo, Japan; 3 Department of Psychiatry, Tokai University School of Medicine, Kanagawa, Japan; 4 Research Center for Child Mental Development, University of Fukui, Fukui, Japan

**Keywords:** Aripiprazole, Autism spectrum disorder, Children and adolescents, Irritability, Post-marketing surveillance

## Abstract

**Background:**

The purpose of this study was to evaluate the post-marketing safety and effectiveness of aripiprazole in treating irritability in pediatric patients (6–17 years) with autism spectrum disorder (ASD) in actual clinical sites of Japan.

**Methods:**

In this post-marketing surveillance, patients were enrolled into the multicenter, prospective, non-interventional, observational study for 52 weeks, and were dosed with aripiprazole (1–15 mg/day) under daily clinical settings in Japan.

**Results:**

In 510 patients, the continuation rate of aripiprazole treatment was 84.6% at day 168 (week 24) and 78.1% at day 364 (week 52). Adverse drug reactions (ADRs) occurred in 22.7% of patients (*n* = 116), and the most common ADRs were somnolence (9.4%), followed by weight increased (3.3%). At week 4, the mean change from baseline in the irritability subscale score for the Aberrant Behavior Checklist Japanese version (ABC-J) was − 5.7 ± 6.8 (*n* = 288). Based on multiple regression analysis, comorbid attention deficit and hyperactivity did not affect the ABC-J irritability subscale score at endpoint. At week 24, the mean change from baseline for the Strengths and Difficulties Questionnaire was − 3.3 ± 4.9 (*n* = 215) for the total difficulties score and 0.6 ± 1.7 (*n* = 217) for the prosocial behavior subscale score.

**Conclusions:**

Aripiprazole was well tolerated and effective in the long-term treatment of irritability associated with ASD in Japanese pediatric patients in the real-world clinical practice.

**Trial registration:**

This surveillance was registered with Clinical Trial.gov (no. NCT03179787) on June 7, 2017 (retrospectively registered).

**Supplementary Information:**

The online version contains supplementary material available at 10.1186/s12888-021-03201-6.

## Background

Autism spectrum disorder (ASD) is characterized by persistent impairment in reciprocal social communication and social interaction, and restricted, repetitive patterns of behavior, interests, or activities. These symptoms are present from early childhood and limit or impair everyday functioning. The impairments in communication and social interaction are pervasive and sustained [1]. Understandably, these symptoms can have a substantial impact on the individuals and their families. This impact can be further increased by the presence of associated behaviors such as irritability, which may manifest as tantrums, aggressiveness, self-injurious behaviors, and sudden mood changes, all of which can have a significant impact on education and social development [2].

Although there are no approved pharmacologic treatments that target core deficits of ASD, associated secondary symptoms such as irritability may be ameliorated by a combination of behavioral and pharmacologic approaches, including the use of atypical antipsychotics [3].

Aripiprazole is an atypical antipsychotic drug developed by Otsuka Pharmaceutical Co., Ltd. that is characterized by partial agonism at dopamine D_2_ receptors and serotonin 5-HT_1A_ receptors and antagonism at 5-HT_2A_ receptors [4, 5]. In the pediatric field, the indications for adolescent schizophrenia (13 to 17 years), pediatric bipolar mania (10 to 17 years), irritability associated with autistic disorder (6 to 17 years), and Tourette’s disorder (6 to 18 years) in the United States, and schizophrenia in adolescents aged 15 years and older in Europe have been approved [6, 7]. Aripiprazole may have a more favorable side-effect profile than other antipsychotics in child and adolescent patients with mental health disorder [8] because of its unique mechanism of action.

The indication for irritability associated with autistic disorder (6 to 17 years) with oral aripiprazole has been approved in the United States since 2009 and in 5 countries thereafter, and the efficacy was also confirmed in clinical trials in children and adolescents with irritability associated with ASD in Japan [9–11]. Based on the trials, the indication for irritability associated with ASD in children and adolescents has been approved since September 2016 in Japan.

The clinical studies that started before 2013 included the patients diagnosed with autistic disorder based on the diagnostic criteria of Diagnostic and Statistical Manual of Mental Disorders, Fourth Edition, Text Revision (DSM-IV-TR) [12]. Before starting this surveillance, DSM-IV-TR had been updated to DSM-5 [1], and the indication was changed to ASD. Since diagnosis with comorbid ASD and attention deficit and hyperactivity disorder (ADHD) was recognized, the scope of applicable patients in this surveillance has been broader than in the clinical study. Also, the criteria for patient selection and concomitant medications are strictly specified in randomized controlled clinical trials and do not necessarily reflect the actual clinical treatment environment. Therefore, this post-marketing surveillance was conducted to assess the real-world safety and effectiveness of aripiprazole for children and adolescents with ASD-related irritability in Japan.

Assessment points for this surveillance were selected based on prior clinical trials in Japan [9–11] and the United States [13–16].

## Methods

### Patients

Patients newly treated with aripiprazole for irritability associated with ASD in children and adolescents (≥6 years and < 18 years) were included in the surveillance. The target sample size was calculated to be 300 patients on the assumption of detecting adverse drug reactions (ADRs) occurring at a frequency of 1% with a confidence of at least 95%.

### Study design

This surveillance was conducted as a multicenter, prospective, non-interventional, observational study for 52 weeks (1 year). The registry and case reports were encoded by the physicians through Electronic Data Capture system during the period from April 2017 to September 2019.

This surveillance was conducted in compliance with the Ministerial Ordinance on Standards for Conducting Post-marketing Surveys and Studies on Drugs; MHLW Ordinance No. 171 issued on December 20, 2004 (GPSP). As the surveillance is a non-interventional study in accordance with GPSP, the need for ethics approval and consent were waived. The surveillance was designed by Otsuka, reviewed by the Japanese Pharmaceutical and Medical Devices Agency (PMDA), and also registered at Clinical Trials.gov (identifier: NCT03179787).

Aripiprazole was administered orally once daily with a starting dose of 1 mg daily and a maintenance dose of 1–15 mg daily according to the package insert in Japan [17]. The dose could be adjusted according to the severity of patient’s symptoms, however the dose increase per day was to be 3 mg or less and the daily dose was not to exceed 15 mg [17].

### Assessments

#### Patient demographics

Patients’ gender, age, body height, weight, severity of symptoms, duration of illness, comorbidities, medical history, concomitant medications, etc. were recorded before aripiprazole administration.

#### Aripiprazole dosing

The daily dose of aripiprazole, administration period (start date and end date), and reasons for discontinuation (if discontinued) were recorded.

#### Safety

The adverse events (if any occurred), onset date, seriousness, outcome, date of outcome, causality to aripiprazole, other possible causal factors, and treatment for adverse events during the observation period were recorded. Seriousness was determined by the physicians.

Body weight and height were also recorded. The shift in the percentile body weight category from baseline to end-point was calculated using predefined body weight percentile categories.

### Effectiveness

Using caregiver-rated Aberrant Behavior Checklist-Japanese version (ABC-J), the degree of aberrant behaviors was scored on four levels ranging from 0 ‘*not at all a problem’* to 3 ‘*the problem is severe in degree*’, totaling 58 items. These items were classified into five subscales: irritability (15 items), lethargy/social withdrawal (16 items), stereotypy (7 items), hyperactivity (16 items), and inappropriate speech (4 items), and scores were calculated [18].

Physician-rated Clinical Global Impression-Improvement (CGI-I) and Severity of illness (CGI-S) scales quantified the physician’s impression at baseline (CGI-S only), week 4, week 8, week 16, week 24, and week 52, or at the time of discontinuation of aripiprazole administration. The CGI-I scale was scored from 1 ‘*very much improved*’ to 7 ‘*very much worse*’, and CGI-S was scored from 1 ‘*normal*’ to 7 ‘*very much severely ill*’.

Caregiver-rated Strengths and Difficulties Questionnaire for Children (SDQ) was used to score the adaptation and mental health conditions on three levels: 0 ‘*not true*’, 1 ‘*somewhat true*’, and 2 ‘*certainly true*’, with a total of 25 items. These items consist of five subscales: conduct problems (5 items), hyperactivity (5 items), emotional problems (5 items), peer problems (5 items), and prosocial behavior (5 items), each yielding scores between 0 and 10. The Total Difficulties Score ranging from 0 to 40 was derived from the sum of the four subscale scores, excluding prosocial behavior [19]. Banding of raw scores obtained with the five SDQ subscales and the Total Difficulties Score, the scale properties were assessed as ‘Low Need’, ‘Some Need’, or ‘High Need’, applying recommended banding of raw scores by Matsuishi T et al. [20] both in ≤13 years and in > 13 years old.

In addition, the average sleep time duration in the last 4 weeks, which was reported by patients or caregivers and not actigraphy-measured data, was recorded at week 24, week 52, and/or end of study. Recommended sleep duration by age group was predefined using modified method reported by Hirshkowitz M et al. [21], and the shift in sleep duration from baseline to end-point (last observation carried forward; LOCF) was calculated.

### Statistical analysis

Results were summarized using descriptive statistics. Continuous variables were described as mean ± standard deviation (SD). The LOCF method was used with imputation of the latest observed values. An applicable paired t-test or Wilcoxon signed-rank sum test was applied to compare the pre-dose and post-dose effect. The level of statistical significance was set at two-sided 5% and the confidence interval at two-sided 95%.

The treatment continuation rate was analyzed by Kaplan-Meier method, with the reason for discontinuation of aripiprazole censored as “transfer,”, “lost to follow-up,” or “improved symptoms”.

The Japanese version of the ICH Medical Dictionary for Regulatory Activities (MedDRA/J version 22.1) was used in coding and classifying adverse events. Adverse events for which a causal relationship to aripiprazole could not be ruled out were considered as ADRs. All statistical analyses were performed using SAS software (version 9.3; SAS Institute Inc.).

## Results

### Patient disposition

**Fig. 1:**
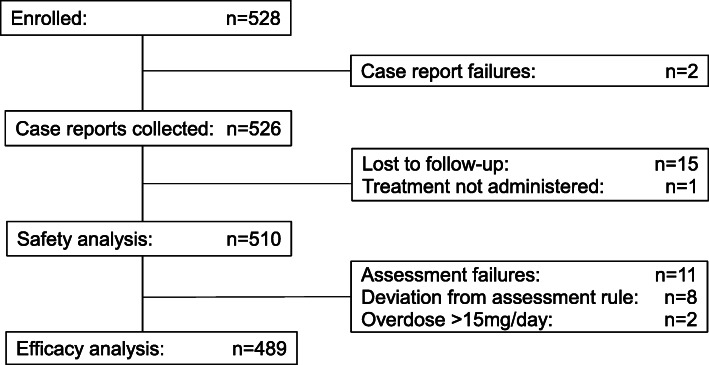
Patient disposition

Five hundred and twenty-eight patients were enrolled at 100 sites across Japan, and 526 case reports were collected during the period of April 2017 to September 2019. The safety analysis population consisted of 510 patients. Among excluded patients, 15 patients were lost to follow-up, and one patient was not treated with aripiprazole. After excluding 21 patients with the protocol deviations (including all effectiveness measures were not assessed after treatment: *n* = 11, all effectiveness measures were assessed over 7 days after last dose: *n* = 7, the daily dose was > 15 mg: *n* = 2, all effectiveness were assessed after day 393: n = 1), the effectiveness analysis population consisted of 489 patients (Fig. 1).

### Patient demographics

Baseline patient characteristics are summarized in Table 1. Among 510 patients included in the safety analysis, the mean age of the patient population at baseline was 10.4 ± 3.1 years, and the majority of the patients were males (73.7%) and aged 6 years or older but younger than 13 years (71.8%).

**Table 1:** Baseline demographics and clinical characteristics (*n* = 510)

	Value, n (%)
Gender	
Male	376 (73.7)
Female	134 (26.3)
Age, years	
6 to 12 years	366 (71.8)
13 to 17 years	144 (28.2)
Mean ± SD	10.4 ± 3.1
Median (min to max)	10.0 (6 to 17)
Treatment Category	
Hospitalization	22 (4.3)
Outpatient	488 (95.7)
Duration of ASD, years	
< 1 year	216 (42.4)
≥ 1 to < 2 years	46 (9.0)
≥ 2 to < 3 years	25 (4.9)
≥ 3 years	86 (16.9)
Unknown	137 (26.9)
Mean ± SD (*n* = 373)	1.70 ± 2.51
Median (min to max)	0.50 (0 to 15.3)
Intellectual disability	
None	387 (75.9)
Total	123 (24.1)
Mild	68 (13.3)
Moderate	30 (5.9)
Severe	19 (3.7)
Most severe	6 (1.2)
Comorbidities	
None	144 (28.2)
Total	366 (71.8)
ADHD^a^	271 (53.1)
LD^a^	57 (11.2)
Tic disorders^a^	35 (6.9)
Sleep disorders^a^	90 (17.6)
Concomitants for ASD	
None	461 (90.4)
Total	49 (9.6)
Risperidone^a^	26 (5.1)
Antidepressants^a^	36 (7.1)
ADHD drugs^a^	6 (1.2)
Concomitants for other condition	
None	257 (50.4)
Total	253 (49.6)
Methylphenidate hydrochloride^ab^	86 (16.9)
Guanfacine hydrochloride^ab^	43 (8.4)
Atomoxetine hydrochloride^ab^	42 (8.2)
Risperidone^ab^	30 (5.9)
Ramelteon^ab^	17 (3.3)
Sodium valproate^ab^	12 (2.4)
Yokukansan^ab^	12 (2.4)
CGI-S	
1: Normal	1 (0.2)
2: Minimally ill	1 (0.2)
3: Mildly ill	42 (8.2)
4: Moderately ill	282 (55.3)
5: Makedly ill	150 (29.4)
6: Severely ill	30 (5.9)
7: Very severely ill	4 (0.8)
Mean ± SD	4.3 ± 0.8
Median (min to max)	4.0 (1 to 7)

a: Multiple responses were allowed in a patient

b: Drugs using in ≥2% of Patiants

Major comorbidities (≥1%) were ADHD in 53.1% (*n* = 271), sleep disorders in 17.6% (*n* = 90), learning disorders (LD) in 11.2% (*n* = 57), and tic disorders in 6.9% (*n* = 35). There were 24.1% (*n* = 123) of patients with intellectual disabilities.

The most common concomitant medications (drugs for ASD irritability, ≥1%) were antidepressants in 7.1% (*n* = 36), risperidone in 5.1% (*n* = 26), and ADHD medications in 1.2% (*n* = 6).

On the physician-rated CGI-S scale, most patients were assessed as being moderately ill (55.3%, *n* = 282), followed by markedly ill (29.4%, *n* = 150).

### Aripiprazole dosing

The mean duration of aripiprazole treatment was 290.6 ± 151.5 days and the mean daily dose was 2.20 ± 1.85 mg.

A total of 201 patients (39.4% of the safety population) discontinued aripiprazole treatment during the surveillance period. Of the 201 patients who discontinued treatment, the most common reason for discontinuation (multiple responses were allowed) was ‘request for discontinuation from the patient or family’ in 33.8% (*n* = 68) (Table 2) and the mean duration of treatment prior to discontinuation was 133.6 ± 113.4 days.

**Table 2:** Reason for discontinuation and the timing

Reason for discontinuation^a^	Timing of discontinuation, n (%)								
	Total	Day 1- < 29	Day 29- < 61	Day 61- < 91	Day 91- < 121	Day 121- < 181	Day 181- < 270	Day 270- < 365	Day ≥365
1. Adverse events	43 (21.4)	12 (6.0)	9 (4.5)	4 (2.0)	4 (2.0)	5 (2.5)	7 (3.5)	2 (1.0)	0 (0.0)
2. Worsening of symptoms	14 (7.0)	2 (1.0)	2 (1.0)	1 (0.5)	1 (0.5)	2 (1.0)	3 (1.5)	3 (1.5)	0 (0.0)
3. Improved symptoms	32 (15.9)	4 (2.0)	1 (0.5)	5 (2.5)	4 (2.0)	3 (1.5)	8 (4.0)	6 (3.0)	1 (0.5)
4. Request for discontinuation from patient or family	68 (33.8)	14 (7.0)	13 (6.5)	5 (2.5)	5 (2.5)	7 (3.5)	13 (6.5)	9 (4.5)	2 (1.0)
5.Transfer to another hospital	21 (10.4)	2 (1.0)	4 (2.0)	0 (0.0)	0 (0.0)	7 (3.5)	4 (2.0)	4 (2.0)	0 (0.0)
6. Lost to follow-up	38 (18.9)	7 (3.5)	7 (3.5)	3 (1.5)	4 (2.0)	5 (2.5)	7 (3.5)	5 (2.5)	0 (0.0)
7. Other	15 (7.5)	2 (1.0)	1 (0.5)	0 (0.0)	2 (1.0)	7 (3.5)	2 (1.0)	0 (0.0)	1 (0.5)
Total^b^	201 (100.0)	37 (18.4)	34 (16.9)	16 (8.0)	17 (8.5)	30 (14.9)	39 (19.4)	24 (11.9)	4 (2.0)

a: Multiple responses were allowed in a patient. Concurrent reasons are: 3 and 4 (*n* = 9), 1 and 4 (*n* = 8), 1 and 2 (*n* = 4), 1 and 7 (*n* = 3), 4 and 7 (*n* = 2), 2 and 4 (*n* = 1), 1 and 6 (*n* = 1), 1, 2, and 4 (*n* = 1)

b: If more than one reason for discontinuation was observed in a patient, the data were summarized as 1 patient

The continuation rate of aripiprazole treatment was 84.6% at day 168 (week 24) and 78.1% at day 364 (week 52) from the first dose (see Additional file 1).

### Safety

**Fig. 2:**
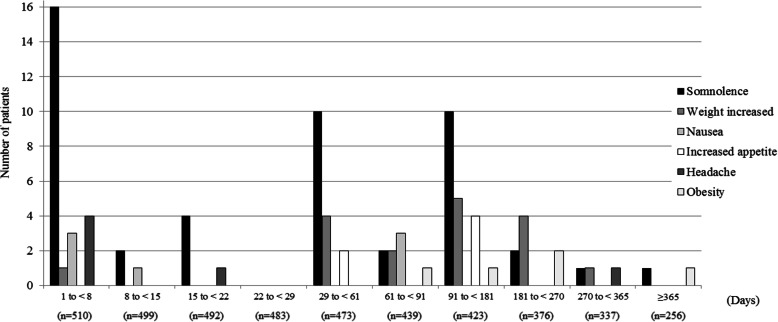
First onset date of adverse drug reactions that occurred in ≥1% of patients

In 510 patients included in the safety analysis, adverse events occurred in 24.5% (*n* = 125) and ADRs based on physician-assessed causality occurred in 22.7% (*n* = 116) (Table 3). The most common ADRs with an incidence of ≥1% were somnolence (9.4%, *n* = 48), followed by weight increased (3.3%, *n* = 17), nausea (1.4%, *n* = 7), increased appetite and headache (1.2% each, *n* = 6), and obesity (1.0%, *n* = 5). Fig. 2 shows the duration to onset date of ADRs from the first dose, and the summary statistics (median [min-max]) were headache (3.5 days [1–284 days]), nausea (8.0 days [2–82 days]), somnolence (32.5 days [1–383 days]), increased appetite (120.5 days [29–179 days]), weight increased (168.0 days [1–320 days]), and obesity (192.0 days [76–365 days]).

**Table 3:** Adverse events and adverse drug reactions occurring in ≥0.5% of patients

	Adverse Events* n = 510	Adverse Drug Reactions* n = 510
	125 (24.5%)	116 (22.7%)
Somnolence	48 (9.4%)	48 (9.4%)
Weight increased	18 (3.5%)	17 (3.3%)
Headache	8 (1.6%)	6 (1.2%)
Nausea	7 (1.4%)	7 (1.4%)
Increased appetite	6 (1.2%)	6 (1.2%)
Obesity	5 (1.0%)	5 (1.0%)
Insomnia	4 (0.8%)	3 (0.6%)
Tic disorders	4 (0.8%)	4 (0.8%)
Akathisia	4 (0.8%)	4 (0.8%)
Constipation	4 (0.8%)	4 (0.8%)
Malaise	4 (0.8%)	4 (0.8%)
Irritability	3 (0.6%)	3 (0.6%)
Dizziness	3 (0.6%)	3 (0.6%)
Vomiting	3 (0.6%)	3 (0.6%)

*: MedDRA/J version (22.1)

Serious ADRs were epilepsy, partial seizures, and renal impairment (0.2% each).

Most common ADRs leading to discontinuation with an incidence of > 0.5% were somnolence (2.0%), weight increased (0.8%), and insomnia (0.6%).

The most common ADRs (≥0.5%) associated with extrapyramidal symptoms were tic disorders and akathisia (0.8% each), both of which were non-serious.

Weight increase-related ADRs occurred in 4.3% of patients (*n* = 22), including weight increased (3.3%) and obesity (1.0%), all of which were non-serious. Among these ADRs, one event led to treatment discontinuation (weight increased, 0.6%).

The mean percentile of weight was 48.36% ± 31.86% at baseline, 52.35% ± 32.86% at end-point (LOCF), and the mean change from baseline to end-point was 3.99% ± 11.65%. Percentile body weight categorical shift data are presented in Table 4. All patients with a weight in the > 90th percentile at end-point (LOCF) were in the > 75th percentile at baseline, and all patients with a weight in the ≤10th percentile at end-point (LOCF) were in the ≤25th percentile at baseline. There were no patients in which the percentiles of weight differed greatly between before and after aripiprazole treatment.

**Table 4:** Shift from baseline to end-point (LOCF) in percentile distribution of body weight

End Point Percentile Category (LOCF)	Baseline Percentile Category, n (%)								
	≤5	> 5- ≤ 10	> 10- ≤ 25	> 25- ≤ 50	> 50- ≤ 75	> 75- ≤ 90	> 90- ≤ 95	> 95	Total
≤5	22 (73.3)	1 (11.1)	1 (2.3)	0	0	0	0	0	24 (9.6)
> 5- ≤ 10	6 (20.0)	3 (33.3)	5 (11.6)	0	0	0	0	0	14 (5.6)
> 10- ≤ 25	1 (3.3)	3 (33.3)	17 (39.5)	8 (17.4)	0	0	0	0	29 (11.6)
> 25- ≤ 50	1 (3.3)	2 (22.2)	19 (44.2)	19 (41.3)	9 (15.0)	0	0	0	50 (19.9)
> 50- ≤ 75	0	0	1 (2.3)	16 (34.8)	29 (48.3)	3 (10.3)	0	0	49 (19.5)
> 75- ≤ 90	0	0	0	3 (6.5)	22 (36.7)	14 (48.3)	3 (20.0)	1 (5.3)	43 (17.1)
> 90- ≤ 95	0	0	0	0	0	9 (31.0)	6 (40.0)	2 (10.5)	17 (6.8)
> 95	0	0	0	0	0	3 (10.3)	6 (40.0)	16 (84.2)	25 (10.0)
Total	30 (100.0)	9 (100.0)	43 (100.0)	46 (100.0)	60 (100.0)	29 (100.0)	15 (100.0)	19 (100.0)	251 (100.0)

### Effectiveness

**Fig. 3:**
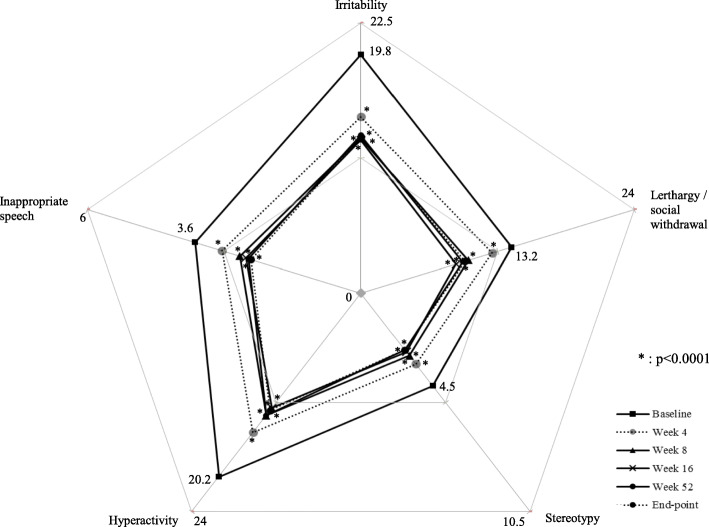
ABC-J score. *Irritability*: 19.8 ± 9.5 at baseline, 14.7 ± 8.8 at Week 4, 12.9 ± 8.1 at Week 8, 12.7 ± 8.3 at Week 16, 13.1 ± 9.1 at Week 52, 13.0 ± 9.0 at end-point (LOCF, n = 396). *Lerthargy / social withdrawal*: 13.2 ± 9.6 at baseline, 11.5 ± 8.9 at Week 4, 9.4 ± 8.5 at Week 8, 8.3 ± 7.8 at Week 16, 8.9 ± 8.4 at Week 52, 9.1 ± 8.4 at end point (LOCF, n = 392). *Stereotypy*: 4.5 ± 4.8 at baseline, 3.4 ± 4.3 at Week 4, 3.0 ± 4.1 at Week 8, 2.8 ± 3.8 at Week 16, 2.7 ± 3.9 at Week 52, 2.7 ± 4.0 at end-point (LOCF, n = 398). *Hyperactivity*: 20.2 ± 11.6 at baseline, 15.3 ± 9.6 at Week 4, 13.4 ± 9.0 at Week 8, 12.6 ± 8.8 at Week 16, 13.4 ± 10.1 at Week 52, 12.9 ± 9.9 at end-point (LOCF, n = 387). *Inappropriate speech*: 3.6 ± 3.1 at baseline, 3.0 ± 2.8 at Week 4, 2.7 ± 2.6 at Week 8, 2.5 ± 2.6 at Week 16, 2.5 ± 2.5 at Week 52, 2.4 ± 2.6 at end-point (LOCF, n = 399)

The mean of ABC-J irritability subscale scores at baseline was 19.8 ± 9.5 (*n* = 396), decreased to 14.7 ± 8.8 (*n* = 288) at week 4 and to 12.9 ± 8.1 (*n* = 316) at week 8, remained at 12.7 to 13.1 at week 16 to 52, and was 13.0 ± 9.0 (n = 396) at end-point (LOCF). Mean changes of ABC-J irritability subscale scores from baseline were − 5.7 ± 6.8 at 4 weeks, − 7.0 to − 8.3 after week 8 and − 6.8 ± 8.3 at end-point (LOCF), with significant reductions in scores compared with baseline at each assessment point (*p* < 0.0001). Patients showed a mean improvement in all ABC-J subscales at all assessment points and end-point (LOCF) compared with baseline (Fig. 3). Interestingly, based on multiple regression analysis, comorbid ADHD was not selected as the variable affecting the ABC-J irritability subscale score at end-point (see Additional file 2).

From the results of the improvement assessment by CGI-I, the percentage of patients scoring 3 ‘minimally improved’ or less was 74.3% (*n* = 306/412) at week 4, 79.4% (*n* = 342/431) to 85.4% (*n* = 239/280) after week 8, and 77.0% (*n* = 376/488) at end-point (LOCF), with an increasing trend throughout the treatment period (see Additional file 3).

**Fig. 4:**
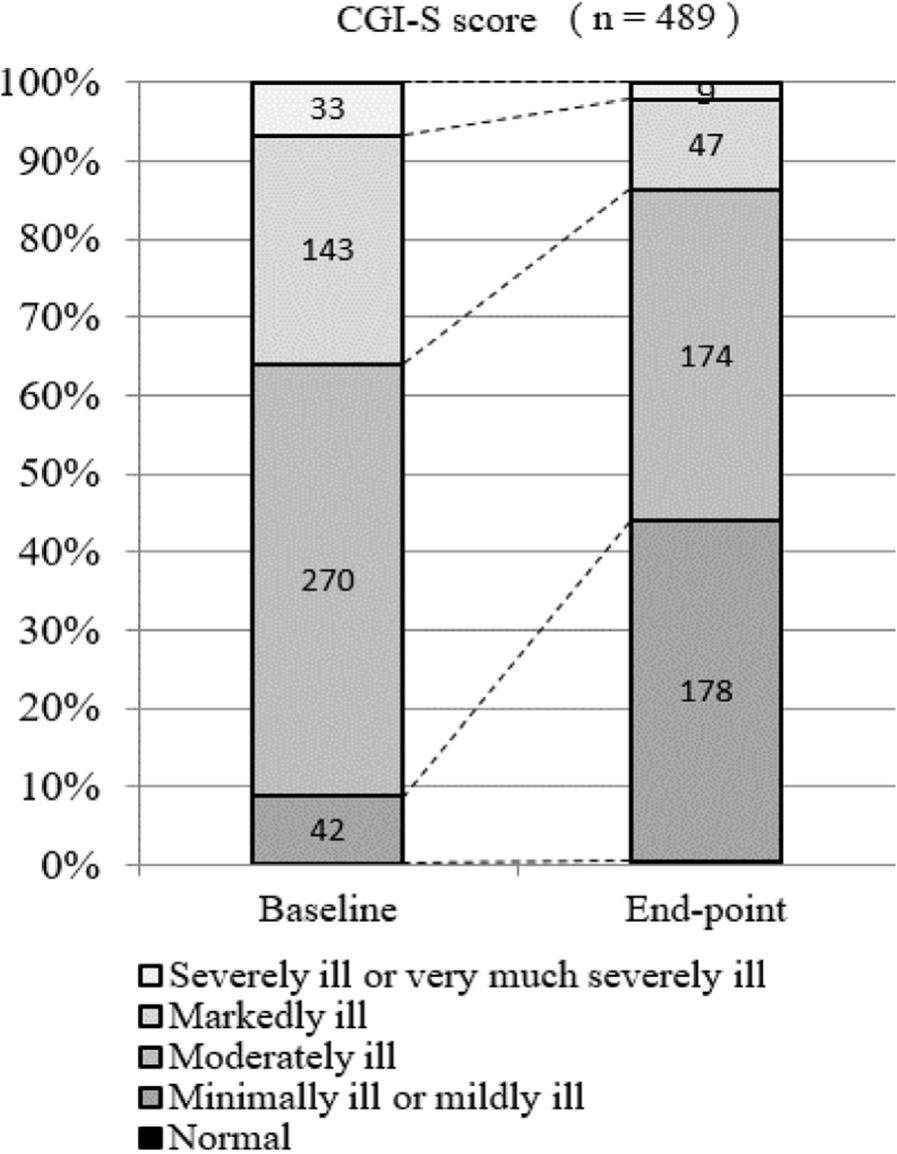
Patient distribution of CGI-S score at baseline and endpoint

From the results of the severity assessment by CGI-S, the percentage of patients who were assessed to be 1 ‘normal’ to 3 ‘mildly ill’ was 8.8% (*n* = 43/489) at baseline, 43.9% (*n* = 180/410) at week 4, 51.3% (*n* = 220/429) at week 8, and 59.7% (*n* = 292/489) at end-point (LOCF) and tended to increase throughout the treatment period (see Fig. 4 and Additional file 3).

**Fig. 5:**
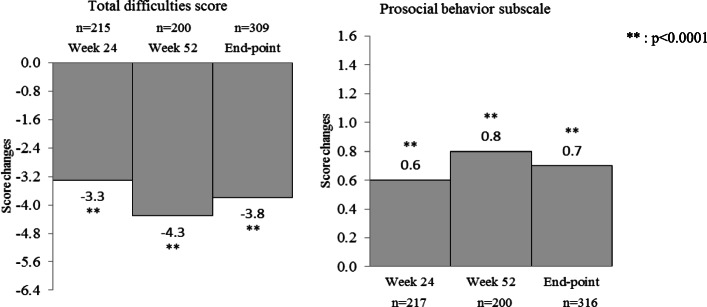
SDQ score. *Total difficulties score*: 21.1 ± 5.5 at baseline, 18.0 ± 5.5 at Week 24, 17.0 ± 6.0 at Week 52, 17.3 ± 5.8 at end-point (LOCF). *Prosocial behavior subscale*: 3.5 ± 2.5 at baseline, 4.2 ± 2.6 at Week 24, 4.3 ± 2.5 at Week 52, 4.3 ± 2.7 at end-point (LOCF)

The mean baseline SDQ Total Difficulties Score was 21.1 ± 5.5 (*n* = 309); mean changes from baseline were − 3.3 ± 4.9 (*n* = 215) at week 24, − 4.3 ± 6.1 (*n* = 200) at week 52, and − 3.8 ± 5.6 (n = 309) at end-point (LOCF) (*p* < 0.0001) (Fig. 5). The mean SDQ prosocial behavior subscale score was 3.5 ± 2.5 (*n* = 316) at baseline, and mean changes from baseline were 0.6 ± 1.7 (*n* = 217) at week 24, 0.8 ± 2.1 (n = 200) at week 52, and 0.7 ± 2.0 (n = 316) at end-point (p < 0.0001) (Fig. 5). All SDQ subscale scores and Total Difficulties Score were significantly decreased at all assessment points compared with baseline. The shift in SDQ scale properties from baseline to end-point (LOCF) is shown in Table 5. For conduct problems, the percentage of patients changing from ‘High Need’ to ‘Some Need’ or ‘Low need’ was 38.8% (*n* = 83/214) and the percentage of patients changing from ‘Some Need’ to ‘Low need’ was 51.1% (*n* = 24/47). In contrast, the percentage of patients changing from ‘Low Need’ to ‘Some Need’ or ‘High need’ was 23.1% (*n* = 12/52) and the patients changing from ‘Some Need’ to ‘High need’ was 19.1% (*n* = 9/47). Also, similar trends were shown in hyperactivity/inattention, emotional symptoms, and prosocial behavior subscales.

**Table 5:** Shift from baseline to end-point (LOCF) in SDQ scale properties

	SDQ scale properties at baseline (bandings of raw scores)	SDQ scale properties at end-point (LOCF), n (%)			
		Low Need	Some Need	High Need	Total
Conduct problems	Low Need (0 to 3)	40 (76.9)	9 (17.3)	3 (5.8)	52 (100.0)
	Some Need (4)	24 (51.1)	14 (29.8)	9 (19.1)	47 (100.0)
	High Need (5 to 10)	44 (20.6)	39 (18.2)	131 (61.2)	214 (100.0)
Hyperactivity/inattention	Low Need (0 to 5)	78 (87.6)	5 (5.6)	6 (6.7)	89 (100.0)
	Some Need (6)	18 (46.2)	13 (33.3)	8 (20.5)	39 (100.0)
	High Need (7 to 10)	56 (29.8)	27 (14.4)	105 (55.9)	188 (100.0)
Emotional symptoms	Low Need (0 to 3)	99 (90.0)	4 (3.6)	7 (6.4)	110 (100.0)
	Some Need (4)	20 (48.8)	9 (22.0)	12 (29.3)	41 (100.0)
	High Need (5 to 10)	43 (25.7)	18 (10.8)	106 (63.5)	167 (100.0)
Peer problems	Low Need (0 to 3)	26 (68.4)	5 (13.2)	7 (18.4)	38 (100.0)
	Some Need (4)	9 (18.4)	25 (51.0)	15 (30.6)	49 (100.0)
	High Need (5 to 10)	22 (9.7)	26 (11.5)	179 (78.9)	227 (100.0)
Total difficulties score	Low Need (0 to 12)	20 (74.1)	3 (11.1)	4 (14.8)	27 (100.0)
	Some Need (13 to 15)	14 (37.8)	16 (43.2)	7 (18.9)	37 (100.0)
	High Need (16 to 40)	45 (18.4)	36 (14.7)	164 (66.9)	245 (100.0)
Prosocial behavior	Low Need (6 to 10)	62 (81.6)	8 (10.5)	6 (7.9)	76 (100.0)
	Some Need (5)	19 (41.3)	18 (39.1)	9 (19.6)	46 (100.0)
	High Need (0 to 4)	29 (14.9)	24 (12.4)	141 (72.7)	194 (100.0)

**Fig. 6:**
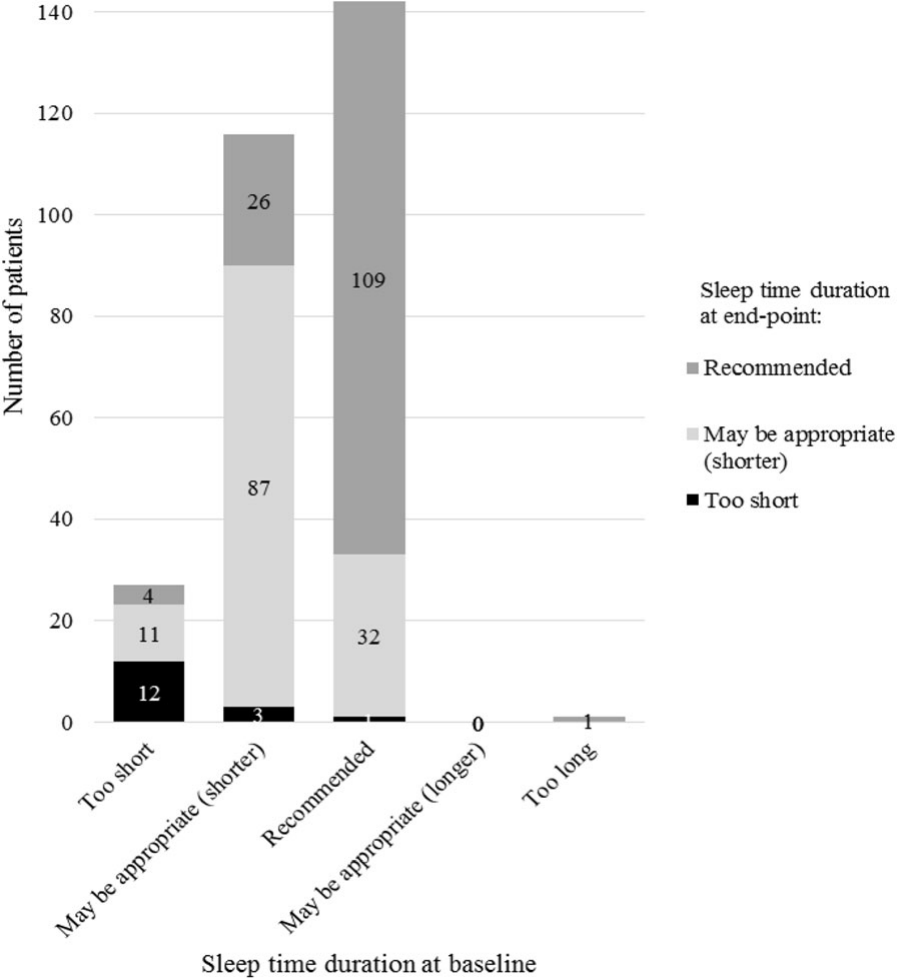
Shift from baseline to end-point (LOCF) in sleep time duration

The shift in mean sleep time duration from baseline to end-point (LOCF) is shown in Fig. 6. The percentage of patients changing from ‘Too short’ to ‘May be appropriate’ or ‘Recommended’ was 55.6% (*n* = 15/27). In contrast, the percentage of patients changing from ‘Recommended’ or ‘May be appropriate’ to ‘Too short’ was 1.6% (*n* = 4/258). Only one patient changed from ‘Too long’ to ‘Recommended’. No patients had a mean sleep time duration of ‘May be appropriate (longer)’ or ‘Too long’ at end-point (LOCF).

## Discussion

Atypical antipsychotics are used to treat irritability, one of the behavioral symptoms associated with ASD in pediatric patients, yet there is a need for additional real-world data on the long-term safety and effectiveness of specific agents. In this surveillance, the safety and effectiveness of aripiprazole in patients (≥6 years to < 18 years old) newly treated with aripiprazole for irritability associated with ASD in children and adolescents were examined in a 52-week observation in daily clinical practice after marketing in Japan.

In the 510 patients included in the safety analysis, the treatment continuation rate was 78.1% at week 52, and the most common reason for discontinuation was ‘request for discontinuation from patient or family’ (33.8%). Treatment continuation might be decided by consultation between the physician and the patient or family members depending on the patient’s conditions or school environment.

The mean daily dose for the entire treatment period in this surveillance was 2.20 ± 1.85 mg, and the mean daily dose of the long-term clinical study was 7.2 ± 4.0 mg [11]. The surveillance cannot be compared with the clinical study because of differences in inclusion/exclusion criteria of patients and dose-escalation/reduction methods. In particular, baseline severity between the short-term clinical study and this surveillance was different; CGI-S scores were 4.9 ± 0.1 (mean ± standard error; SE) and 4.3 ± 0.8, and ABC-J irritability subscale scores were 26.9 ± 1.0 (mean ± SE) and 19.7 ± 9.6, respectively [9], aligning with our findings that aripiprazole was administered to patients with milder symptoms in routine practice than in the clinical study.

In 510 patients included in the safety analysis, ADRs occurred in 22.7% (*n* = 116/510) and the events that occurred in ≥1% of patients were somnolence (9.4%), weight increased (3.3%), nausea (1.4%), increased appetite and headache (1.2% each), and obesity (1.0%). Among these, headache and nausea were observed more frequently in the early phase of treatment, and there was no tendency for increased incidence with long-term treatment. Somnolence was observed through the surveillance period, and there were no reports of fall or traumatic injury related to somnolence. However, since the event is considered to affect school-aged pediatric patients’ academic performance, monitoring of the events is needed.

In contrast, the incidence of treatment-emergent adverse events (TEAEs) was 97.7% (*n* = 84/86) in the Japanese clinical study, and the major events were somnolence (32.6%), influenza (29.1%) and weight increased (24.2%) [11]. Also, the major TEAEs were weight increased (23.0%) and vomiting (18.8%) in American 52-week clinical study [15]. Although the backgrounds of patients, the frequency of observations, and the dose differed from those in the clinical study, somnolence and weight increased were also the most common events in the real-world treatment.

For the effect on weight and growth, 0.6% of weight increase-related ADRs (weight increased and obesity) led to treatment discontinuation, and this was considered not to have a major effect on long-term treatment continuation. The mean change in body weight from baseline increased over time throughout the treatment period, but there were no major changes in weight percentile category from baseline, suggesting that weight increase is due to natural growth and not a crucial safety issue. The percentile and z-score of height and body mass index (BMI) are also suggesting that (see Additional file 4).

ABC-J irritability subscale score was 19.8 ± 9.5 (*n* = 396) at baseline, and the mean changes from baseline were − 5.7 ± 6.8 at week 4 and − 6.8 ± 8.3 at end-point, which were significantly decreased (*p* < 0.0001). The corresponding score was 26.9 ± 1.0 (mean ± SE) at the baseline of the short-term clinical study and 17.6 ± 10.0 at the baseline of the long-term clinical study in Japan. The mean changes from baseline were − 11.4 ± 1.3 (mean ± SE) at week 8 in the short-term clinical study, and − 3.2 ± 8.1 at end-point in the long-term clinical study in Japan [9–11]. Also, the mean change from baseline was − 6.5 ± 11.1 at end-point in American 52-week clinical study [16]. In these clinical studies, enrolled patients with ABC-J irritability subscale score of 18 or higher cannot be directly compared with the surveillance because of differences in patients and treatment characteristics. However, ABC-J irritability subscale score showed that aripiprazole improved the symptoms even in patients with relatively mild symptoms who were treated in post-marketing setting.

Interestingly, although patients with comorbid ADHD were excluded in the clinical trials under diagnosis based on DSM-IV-TR, 53.1% of patients in this surveillance had comorbidities of ADHD that did not affect the ABC-J irritability subscale score at end-point (LOCF).

In addition, overall aberrant behaviors including hyperactivity, stereotypy, inappropriate speech, and lethargy/social withdrawal were improved concurrently with irritability.

SDQ for children has not been assessed in the clinical trials [11], while total 25 items in SDQ are recorded by caregivers in this surveillance. ASD diagnosis has been associated with the low scores on the prosocial subscale in UK cohorts [22], and social impairments are core deficits [23]. Our findings suggest that prosocial behavior, which refers to positive interactions with other people, including helping, sharing, cooperating, and comforting, may also be improved in school based settings from a social perspective as well as irritability symptoms.

Sleep duration has not been measured in prior clinical trials, whereas adequate sleep duration may not be maintained sufficiently due to irritability in the patients with ASD. The average sleep time duration in the last 4 weeks was reported by patients or caregivers and assessed in consideration of the recommended sleep time for each age [21] in this surveillance. Although concomitant use of hypnotics was not taken into account, the improvement of irritability might lead to proper sleep duration as shown in Fig. 6. The results and interpretation of sleep time duration have certain limitations as there are no actigraphy-measured data.

To our knowledge, this is the first study assessing the tolerability and effectiveness of aripiprazole in pediatric patients with ASD associated irritability over the long term, collecting a large amount of data and under actual clinical practice settings, not only for ABC-J or CGI scale but also SDQ scale, even though there are reports of clinical trials including systematic review and meta-analysis [24–28]. The goals of treatment are mainly to maximize an individual’s functional independence and quality of life through development and learning, improvements in social skills and communication, reductions in disability and comorbidity, and promotion of independence [29]. The results of ABC-J and SDQ assessments suggested that aripiprazole improved the symptoms of irritability associated with ASD, which may have a secondarily effect in addressing these treatment goals.

The results and interpretation of safety and effectiveness have certain limitations. This surveillance was a prospective study in which the evaluation methods and measurement scales were determined in advance, and it was an observational study in daily clinical practice without a comparison group. In addition, there were variations in the reporting and evaluation and deficiencies in the assessments based on the reports made by the physicians and caregivers.

## Conclusions

The results of the 52-week post-marketing surveillance suggest that aripiprazole was well tolerated and effective in the long-term treatment of irritability associated with ASD in Japanese children and adolescents in the real-world clinical practice.

## Supplementary Information

**Additional file 1.** Treatment continuation rate (Kaplan-Meier plot).

**Additional file 2.** Factors affecting ABC-J irritability subscale.

**Additional file 3.** Patient distribution of CGI-I and CGI-S score.

**Additional file 4.** Changes from baseline in height, weight and BMI.

## Data Availability

The data that support the findings of this study are available from Clinical Trial.gov (no. NCT03179787).

## Abbreviations

ABC-J: Aberrant Behavior Checklist Japanese version
ADHD: Attention Deficit and Hyperactivity Disorder
ADR: Adverse Drug Reaction
ASD: Autism Spectrum Disorder
BMI: Body Mass Index
CGI-I: Clinical Global Impression-Improvement
CGI-S: Clinical Global Impression-Severity of illness
DSM-IV-TR: Diagnostic and Statistical Manual of Mental Disorders, Fourth Edition, Text Revision
LD: Learning Disorders
LOCF: Last Observation Carried Forward
MedDRA/J: Japanese version of the ICH Medical Dictionary for Regulatory Activities
PMDA: Japanese Pharmaceutical and Medical Devices Agency
SD: Standard Deviation
SDQ: Strengths and Difficulties Questionnaire for Children
SE: Standard Error
TEAE: Treatment-emergent Adverse Event

## References

[1] American Psychiatric Association (2013). Diagnostic and Statistical Manual of Mental Disorders, 5th edn.

[2] Volkmar F, Cook EH, Pomeroy J, Realmuto G, Tanguay P (1999). Practice parameters for the assessment and treatment of children, adolescents, and adults with autism and other pervasive developmental disorders. American Academy of Child and Adolescent Psychiatry working group on quality issues. J Am Acad Child Adolesc Psychiatry.

[3] Myers SM, Johnson CP (2007). American Academy of Pediatrics Council on children with disabilities. Management of children with autism spectrum disorders. Pediatrics..

[4] Tadori Y, Kitagawa H, Forbes RA, McQuade RD, Stark A, Kikuchi T (2007). Differences in agonist/antagonist properties at human dopamine D_2_ receptors between aripiprazole, bifeprunox and SDZ 208-912. Eur J Pharmacol.

[5] Stark AD, Jordan S, Allers KA, Bertekap RL, Chen R, Mistry Kannan T, et al. Interaction of the novel antipsychotic aripiprazole with 5-HT1A and 5-HT 2A receptors: functional receptor binding and in vivo electrophysiological studies. Psychopharmacology. 2007;190(3):373–82. 10.1007/s00213-006-0621-y. 10.1007/s00213-006-0621-y 17242925

[6] Otsuka America Pharmaceutical, Inc. Aripiprazole [package insert]. Rockville; 2020. https://www.accessdata.fda.gov/scripts/cder/safetylabelingchanges/.

[7] Otsuka Pharmaceutical Netherlands B.V. Aripiprazole [summary of product characteristics]. Amsterdam; 2020. https://www.ema.europa.eu/en/medicines/human/EPAR/abilify.

[8] Caccia S (2013). Safety and pharmacokinetics of atypical antipsychotics in children and adolescents. Pediatr Drugs.

[9] Ichikawa H, Mikami K, Okada T, Yamashita Y, Ishizaki Y, Tomoda A, et al. Aripiprazole in the treatment of irritability in children and adolescents with autism spectrum disorder in Japan: a randomized, double-blind, placebo-controlled study. Child Psychiat Hum D. 2017;48(5):796–806. 10.1007/s10578-016-0704-x. 10.1007/s10578-016-0704-x PMC5617873 28004215

[10] Ichikawa H, Hiratani M, Yasuhara A (2016). An open-label extension study of the safety and efficacy of aripiprazole for irritability in children and adolescents with autistic disorder: an interim report. Jpn J Clin Psychopharmacol.

[11] Ichikawa H, Hiratani M, Yasuhara A, Tsujii N, Oshimo T, Ono H, et al. An open-label extension long-term study of the safety and efficacy of aripiprazole for irritability in children and adolescents with autistic disorder in Japan. Psychiatry Clin Neurosci. 2018;72(2):84–94. 10.1111/pcn.12607. 10.1111/pcn.12607 28941259

[12] American Psychiatric Association (2000). Diagnostic and Statistical Manual of Mental Disorders, 4th edn, text revision edn.

[13] Owen R, Sikich L, Marcus RN, Corey-Lisle P, Manos G, McQuade RD (2009). Aripiprazole in the treatment of irritability in children and adolescents with autistic disorder. Pediatrics..

[14] Marcus RN, Owen R, Kamen L, Manos G, McQuade RD, Carson WH, et al. A placebo-controlled, fixed-dose study of aripiprazole in children and adolescents with irritability associated with autistic disorder. J Am Acad Child Adolesc Psychiatry. 2009;48(11):1110–9. 10.1097/CHI.0b013e3181b76658. 10.1097/CHI.0b013e3181b76658 19797985

[15] Marcus RN, Owen R, Manos G, Mankoski R, Kamen L, McQuade RD (2011). Safety and tolerability of aripiprazole for irritability in pediatric patients with autistic disorder: a 52-week, openlabel, multicenter study. J Clin Psychiatry.

[16] Marcus RN, Owen R, Manos G, Mankoski R, Kamen L, McQuade RD (2011). Aripiprazole in the treatment of irritability in pediatric patients (aged 6-17 years) with autistic disorder: results from a 52-week, open-label study. J Child Adolesc Psychopharmacol.

[17] Otsuka Pharmaceutical Co., Ltd. Aripiprazole [package insert]. Tokyo, Japan, Revised September 2020 (in Japanese).

[18] Ono Y (1996). Factor validity and reliability for the aberrant behavior checklist-Community in a Japanese population with mental retardation. Res Dev Disabil.

[19] Goodman R (1997). The strengths and difficulties questionnaire: a research note. J Child Psychol Psychiatry.

[20] Matsuishi T, Nagano M, Araki Y, Tanaka Y, Iwasaki M, Yamashita Y, et al. Scale properties of the Japanese version of the strengths and difficulties questionnaire (SDQ): a study of infant and school children in community samples. Brain and Development. 2008;30(6):410–5. 10.1016/j.braindev.2007.12.003. 10.1016/j.braindev.2007.12.003 18226867

[21] Hirshkowitz M, Whiton K, Albert SM, Alessi C, Bruni O, DonCarlos L, et al. National Sleep Foundation’s sleep time duration recommendations: methodology and results summary. Sleep Health. 2015;1(1):40–3. 10.1016/j.sleh.2014.12.010. 10.1016/j.sleh.2014.12.010 29073412

[22] Russell G, Steer C (2011). Golding J social and demographic factors that influence the diagnosis of autistic spectrum disorders. Soc Psychiatry Psychiatr Epidemiol.

[23] Iizuka C, Yamashita Y, Nagamitsu S, Yamashita T, Araki Y, Ohya T, et al. Comparison of the strengths and difficulties questionnaire (SDQ) scores between children with high-functioning autism spectrum disorder (HFASD) and attention-deficit/hyperactivity disorder (AD/HD). Brain and Development. 2010;32(8):609–12. 10.1016/j.braindev.2009.09.009. 10.1016/j.braindev.2009.09.009 19828270

[24] Robb AS, Andersson C, Bellocchio EE, Manos G, Rojas-Fernandez C, Mathew S, et al. Safety and tolerability of aripiprazole in the treatment of irritability associated with autistic disorder in pediatric subjects (6-17 years old):results from a pooled analysis of 2 studies. Prim Care Companion CNS Disord. 2011;13(1):1–9. 10.4088/PCC.10m01008gry. 10.4088/PCC.10m01008gry PMC3121213 21731831

[25] Mankoski R, Stockton G, Manos G, Marler S, McQuade R, Forbes RA, et al. Aripiprazole treatment of irritability associated with autistic disorder and the relationship between prior antipsychotic exposure, adverse events, and weight change. J Child Adolesc Psychopharmacol. 2013;23(8):572–6. 10.1089/cap.2012.0075. 10.1089/cap.2012.0075 PMC3804231 24138011

[26] Coustals N, Menard ML, Cohen D (2021). Aripiprazole in children and adolescents. J Child Adolesc Psychopharmacol..

[27] Maneeton N, Maneeton B, Putthisri S, Suttajit S, Likhitsathian S, Srisurapanont M (2018). Aripiprazole in acute treatment of children and adolescents with autism spectrum disorder: a systematic review and metaanalysis. Neuropsychiatr Dis Treat.

[28] Hirsch LE, Pringsheim T. Aripiprazole for autism spectrum disorders (ASD). Cochrane Database Syst Rev. 2016;(6). Art. No.: CD009043. 10.1002/14651858.CD009043.pub3. 10.1002/14651858.CD009043.pub3 PMC7120220 27344135

[29] Lai MC, Lombardo MV, Baron-Cohen S (2014). Autism. Lancet..

