# Supplementary material for: Aripiprazole in the real-world treatment for irritability associated with autism spectrum disorder in children and adolescents in Japan: 52-week post-marketing surveillance
Source: BMC Psychiatry. 2021 Apr 22;21:204. doi: 10.1186/s12888-021-03201-6 (PMC8061053; doi:10.1186/s12888-021-03201-6)

Aripiprazole in the real-world treatment for irritability associated with autism spectrum disorder in children and adolescents in Japan: 52-week post-marketing surveillance

Yuna Sugimoto[1*]; Kayo Yamamura[1]; Tomoyo Takayama[1]; Yasuhiko Fukuta[1]; Kazuo Aoki[2], Katsunaka Mikami[3]; Akemi Tomoda[4]

[*]*Correspondence: Sugimoto.Yuna@otsuka.jp*

[1]Pharmacovigilance Department, Otsuka Pharmaceutical Co., Ltd., Tokyo, Japan

[2]Medical Affairs Department, Otsuka Pharmaceutical Co., Ltd., Tokyo, Japan.

[3]Department of Psychiatry, Tokai University School of Medicine, Kanagawa, Japan.

[4]Research Center for Child Mental Development, University of Fukui, Fukui, Japan.

Additional file 1. Treatment continuation rate (Kaplan-Meier plot)

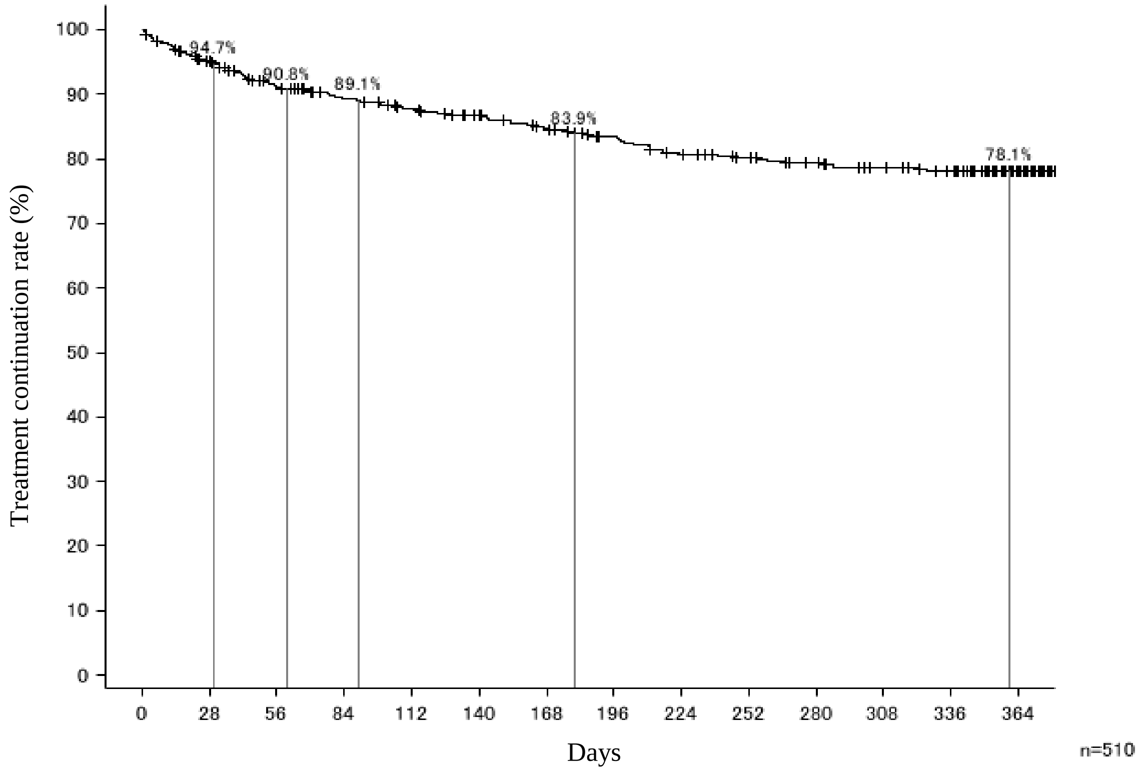

Supplement: Supplementary file 1 — Additional file 1. Treatment continuation rate (Kaplan-Meier plot). [file 12888_2021_3201_MOESM1_ESM.pdf]
