# Supplementary material for: Aripiprazole in the real-world treatment for irritability associated with autism spectrum disorder in children and adolescents in Japan: 52-week post-marketing surveillance
Source: BMC Psychiatry. 2021 Apr 22;21:204. doi: 10.1186/s12888-021-03201-6 (PMC8061053; doi:10.1186/s12888-021-03201-6)
Supplement: Supplementary file 2 — Additional file 2. Factors affecting ABC-J irritability subscale. [file 12888_2021_3201_MOESM2_ESM.pdf]

Additional file 2. Factors affecting ABC-J irritability subscale

Factors		Multivariate [a]		
		Point estimation	95% confidence interval	P-value
Age		-	-	-
Age at diagnosis of ASD		-0.23095	-0.45527--0.00663	p=0.0436*
ABC-J irritability subscale score at baseline		0.54883	0.45836-0.63929	p<0.0001**
CGI-S score at baseline		-	-	-
Mean daily dose		-	-	-
Mean duration of aripiprazole treatment		-0.00609	-0.01279-0.00062	p=0.0749
Gender	Male	-	-	-
	Female	-	-	-
Intellectual disability	None	-	-	-
	Mild	-	-	-
	Moderate	-	-	-
	Severe	-	-	-
	Most severe	-	-	-
Comorbidities	None	-	-	-
	ADHD	-	-	-
Concomitants	None	-	-	-
	Antipsychotic	-	-	-

a: Covariance analysis model. Factors that were not selected are shown in hyphens. ** : p<0.01, * : p<0.05

Response variable : ABC-J irritability subscale score at end-point

Explanatory variable : Variable selection was performed using the variable increase / decrease method (stepwise) from the following patient backgrounds that are thought to have a clinical effect on effectiveness evaluation. The level of variable selection was 0.15. If an item with an unknown or missing item was selected as an explanatory variable, cases with an item classified as unknown or missing were excluded.

Continuous variable : Age, age at diagnosis of ASD, baseline ABC-J irritability subscale score, baseline CGI-S score, mean daily dose, mean duration of aripiprazole treatment

Discrete variable : Gender, intellectual disability, comorbidities, concomitants
