# Supplementary figures and images for: Aripiprazole in the real-world treatment for irritability associated with autism spectrum disorder in children and adolescents in Japan: 52-week post-marketing surveillance
Source: BMC Psychiatry. 2021 Apr 22;21:204. doi: 10.1186/s12888-021-03201-6 (PMC8061053; doi:10.1186/s12888-021-03201-6)

Additional file 3. Patient distribution of CGI-I and CGI-S score

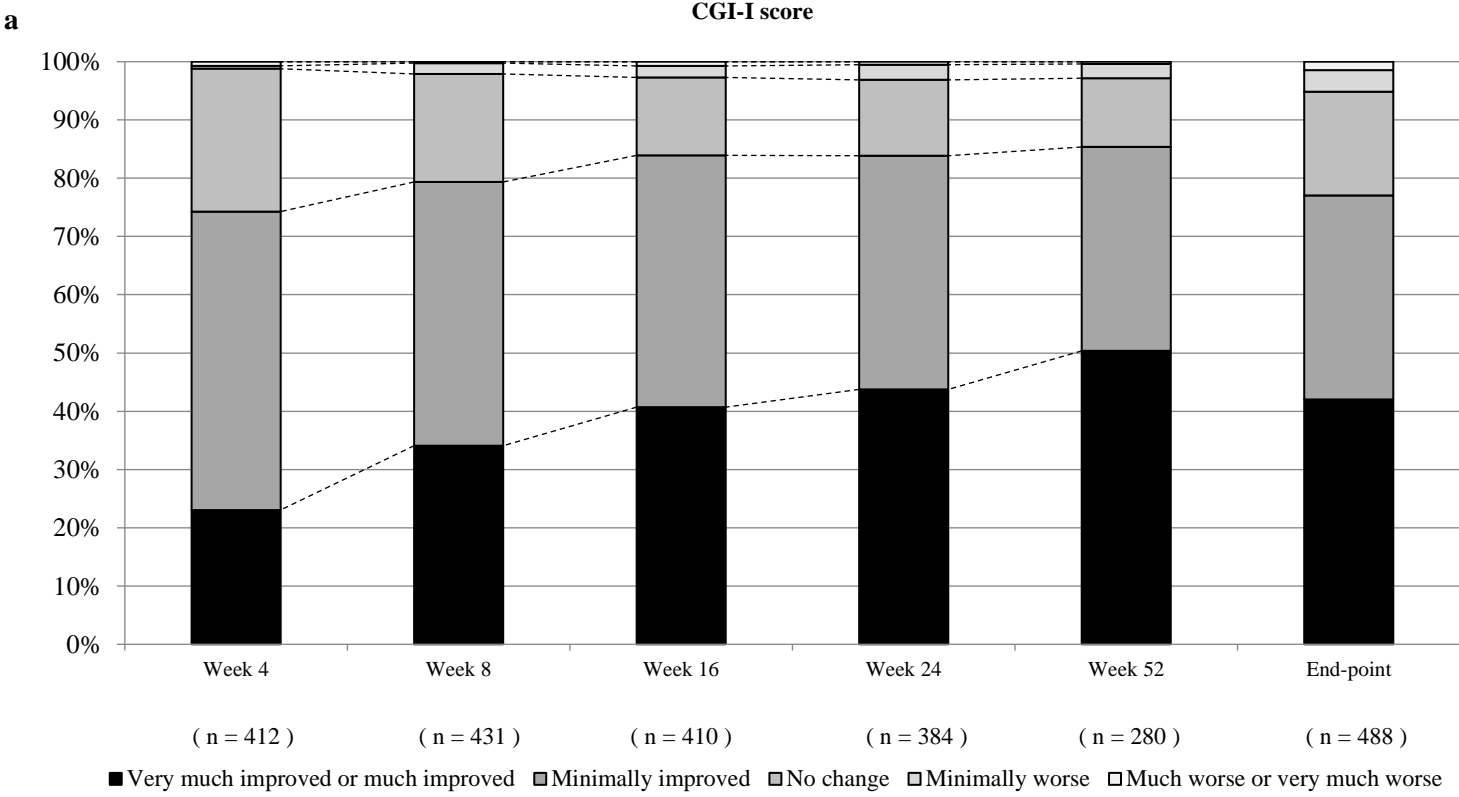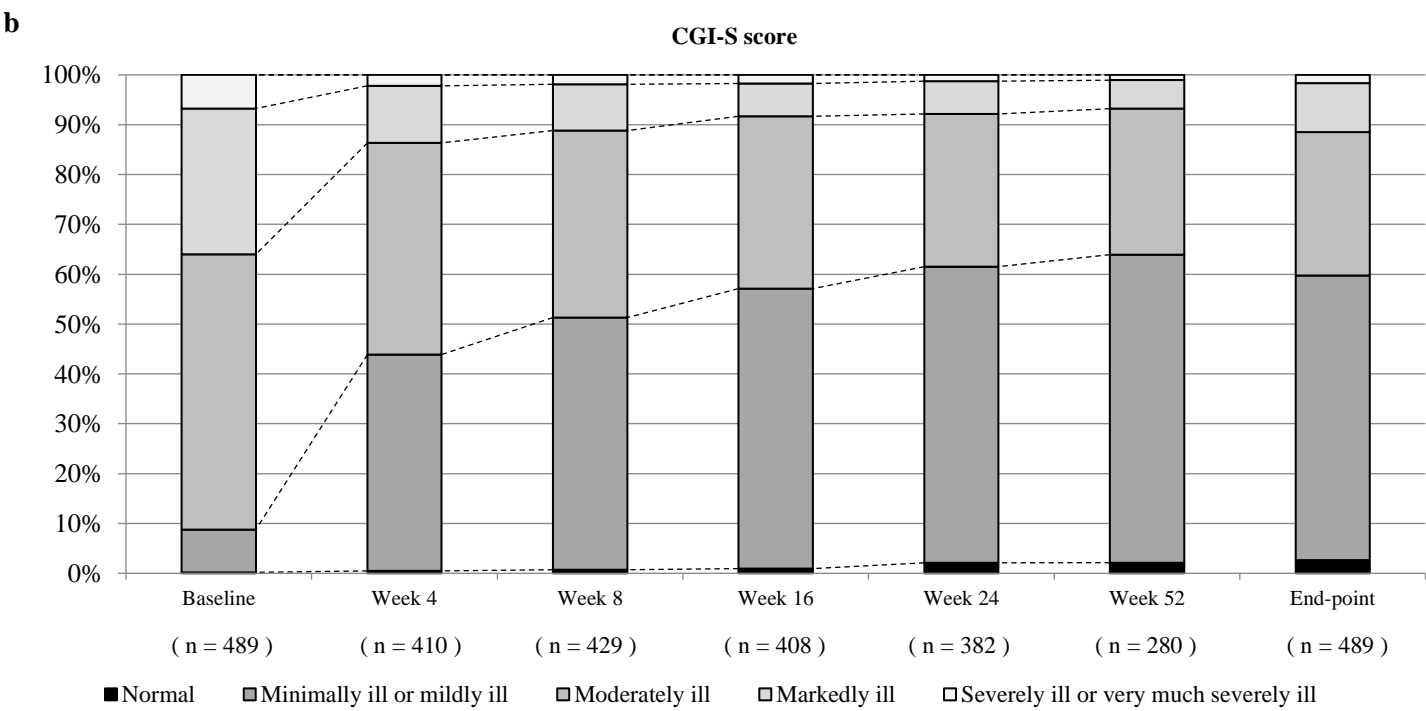

Supplement: Supplementary file 3 — Additional file 3. Patient distribution of CGI-I and CGI-S score. [file 12888_2021_3201_MOESM3_ESM.pdf]
