# Supplementary material for: Aripiprazole in the real-world treatment for irritability associated with autism spectrum disorder in children and adolescents in Japan: 52-week post-marketing surveillance
Source: BMC Psychiatry. 2021 Apr 22;21:204. doi: 10.1186/s12888-021-03201-6 (PMC8061053; doi:10.1186/s12888-021-03201-6)
Supplement: Supplementary file 4 — Additional file 4. Changes from baseline in height, weight and BMI. [file 12888_2021_3201_MOESM4_ESM.pdf]

Additional file 4. Changes from baseline in height, weight and BMI

		Baseline			End-point (LOCF)			Change from baseline		
		n	mean	SD	n	mean	SD	n	mean	SD
< 13 years old	Height (cm)	179	131.72	13.27	179	136.58	13.26	179	4.86	2.79
	Height z-score	179	-0.20	1.10	179	-0.19	1.07	179	0.01	0.39
	Height percentile	179	45.34	29.96	179	46.00	29.57	179	0.66	12.24
	Weight (kg)	195	30.98	11.48	195	35.34	13.16	195	4.36	3.53
	Weight z-score	195	-0.07	1.18	195	0.09	1.23	195	0.16	0.45
	Weight percentile	195	48.76	31.21	195	53.25	32.25	195	4.49	12.25
	BMI (kg/m^2)	178	17.49	3.66	178	18.54	4.14	178	1.05	1.37
	BMI z-score	178	0.05	1.10	178	0.23	1.20	178	0.18	0.58
	BMI percentile	178	50.65	30.31	178	56.25	31.51	178	5.60	15.22
≥13 years old	Height (cm)	52	158.83	8.12	52	160.68	7.75	52	1.85	2.19
	Height z-score	52	-0.37	1.10	52	-0.40	1.14	52	-0.03	0.27
	Height percentile	52	40.13	30.89	52	39.32	31.25	52	-0.81	7.05
	Weight (kg)	56	52.61	12.02	56	56.53	13.38	56	3.92	4.61
	Weight z-score	56	-0.15	1.34	56	0.01	1.36	56	0.16	0.42
	Weight percentile	56	46.99	34.30	56	49.25	35.03	56	2.26	9.14
	BMI (kg/m^2)	51	21.15	4.15	51	22.16	4.85	51	1.01	1.63
	BMI z-score	51	0.10	1.23	51	0.26	1.25	51	0.16	0.42
	BMI percentile	51	52.18	33.02	51	55.30	32.79	51	3.12	12.71
Total	Height (cm)	231	137.83	16.72	231	142.01	15.85	231	4.18	2.95
	Height z-score	231	-0.24	1.10	231	-0.23	1.09	231	0.00	0.37
	Height percentile	231	44.17	30.18	231	44.50	30.02	231	0.33	11.28
	Weight (kg)	251	35.81	14.68	251	40.07	15.87	251	4.27	3.79
	Weight z-score	251	-0.09	1.21	251	0.08	1.26	251	0.16	0.44
	Weight percentile	251	48.36	31.86	251	52.35	32.86	251	3.99	11.65
	BMI (kg/m^2)	229	18.30	4.06	229	19.35	4.55	229	1.04	1.43
	BMI z-score	229	0.06	1.13	229	0.23	1.21	229	0.17	0.55
	BMI percentile	229	50.99	30.87	229	56.04	31.73	229	5.05	14.70
